# Notch1-induced T cell leukemia can be potentiated by microenvironmental cues in the spleen

**DOI:** 10.1186/s13045-014-0071-7

**Published:** 2014-11-04

**Authors:** Shihui Ma, Yingxu Shi, Yakun Pang, Fang Dong, Hui Cheng, Sha Hao, Jing Xu, Xiaofan Zhu, Weiping Yuan, Tao Cheng, Guoguang Zheng

**Affiliations:** State Key Laboratory of Experimental Hematology, Institute of Hematology and Blood Diseases Hospital, Chinese Academy of Medical Sciences and Peking Union Medical College, Tianjin, 300020 China; Center for Stem Cell Medicine, Chinese Academy of Medical Sciences, Beijing, 100730 China; Current address of Yingxu Shi: Affiliated Hospital Clinical Laboratory, Inner Mongolian Medical University, Hohhot, China

**Keywords:** Spleen, Microenvironment, T cell acute lymphoblastic leukemia, MIP-3β, Splenectomy

## Abstract

**Background:**

Leukemia is a systemic malignancy originated from hematopoietic cells. The extracellular environment has great impacts on the survival, proliferation and dissemination of leukemia cells. The spleen is an important organ for extramedullary hematopoiesis and a common infiltration site in lymphoid malignancies. Splenomegaly, frequently observed in T cell acute lymphoblastic leukemia (T-ALL), is associated with poor prognosis. However, how the spleen microenvironment distinctly affects T-ALL cells as opposed to bone marrow (BM) microenvironment has not been addressed.

**Methods:**

A Notch1-induced mouse T-ALL model was applied in this study. Flow cytometry and two-photon fluorescence microscopy were used to analyze early distribution of T-ALL cells. MILLIPLEX® MAP Multiplex Immunoassay was performed to measure cytokine/chemokine levels in different microenvironments. Transwell and co-culture experiments were used to test the effects of splenic microenvironment in vitro. Splenectomy was performed to assess the organ specific impact on the survival of T-ALL-bearing mice.

**Results:**

More leukemia cells were detected in the spleen than in the BM after injection of T-ALL cells by flow cytometry and two-photon fluorescence microscopy analysis. By screening a panel of cytokines/chemokines, a higher level of MIP-3β was found in the splenic microenvironment than BM microenvironment. In vitro transwell experiment further confirmed that MIP-3β recruits T-ALL cells which express a high level of MIP-3β receptor, CCR7. Furthermore, the splenic microenvironment stimulates T-ALL cells to express a higher level of MIP-3β, which further recruits T-ALL cells to the spleen. Co-culture experiment found that the splenic microenvironment more potently stimulated the proliferation and migration of T-ALL cells than BM. Moreover, the mice transplanted with T-ALL cells from the spleen had a shorter life span than those transplanted from BM, suggesting increased potency of the T-ALL cells induced by the splenic microenvironment. In addition, splenectomy prolonged the survival of leukemic mice.

**Conclusions:**

Our study demonstrates an organ specific effect on leukemia development. Specifically, T-ALL cells can be potentiated by splenic microenvironment and thus spleen may serve as a target organ for the treatment of some types of leukemia.

**Electronic supplementary material:**

The online version of this article (doi:10.1186/s13045-014-0071-7) contains supplementary material, which is available to authorized users.

## Background

The microenvironment plays important roles in the survival and function of cells [1]. Mounting evidence suggests that an abnormal environment not only promotes the malignant transformation of cells but also has great impacts on the development of malignancies [2,3]. For example, the mesenchymal-specific deletion of CSL/RBP-Jk, a key Notch effector, is sufficient to induce several features associated with field cancerization in the skin [4]. Moreover, the deletion of Dicer1 in mouse osteo-lineage cells in a specific hematopoietic niche can result in myelodysplasia; mice with this alteration eventually develop myelodysplastic syndrome (MDS) and secondary leukemia [5]. In addition, tumor microenvironments are mostly beneficial to malignant cells but not to their normal cell counterparts; these microenvironments therefore contribute to the spreading and outgrowth of malignant cells [6,7]. This paradigm is best exemplified in the hematopoietic system.

Normal hematopoiesis is strictly regulated by intracellular and environmental factors. Hematopoietic stem cells (HSCs) and hematopoietic progenitor cells (HPCs) are believed to reside in the specific hematopoietic microenvironments known as HSC/HPC niches; several niches for HSCs have been proposed [8-10]. The dysfunction of niches for HSCs and HPCs also contributes to hematopoietic disorders [11]. The initiation of leukemia is caused by both intrinsic factors, such as the aberrant expression of oncogenes or tumor suppressors [12,13], and extrinsic factors, such as immune dysfunction, neovasculature and other tumor-promoting microenvironmental cues in the hematopoietic system [14]. As a clonal disease, leukemia cells must compete with normal hematopoietic cells to become the predominant cell population. During the development of leukemia, leukemia cells induce a leukemic environment that is favorable for the outgrowth of leukemia cells and dissemination of leukemia cells to hematopoietic organs and tissues [6,15], primarily via blood circulation. The bone marrow (BM) niche for leukemia cells has also been suggested to play important roles in the survival, growth and malignant phenotypes of leukemia cells, at least during the early stages of leukemia [16].

Cytokines and chemokines are important components of the cell microenvironment and have profound effects on both normal and malignant cells. For example, a high level of stem cell factor (SCF) has been detected in mouse leukemia models [6,17]. It has also been suggested that leukemia cells force normal HSCs and HPCs out of their BM niches and occupy these niches by secreting SCF [6]. Chemokines, which are classified into four main subfamilies, CXC, CC, CX3C and XC, are a family of small cytokines or signaling proteins that are secreted by cells. They act as chemoattractants to guide the migration of cells by interacting with G protein-linked transmembrane receptors [18,19]. Macrophage inflammatory protein (MIP)-3β, also known as CCL19, is a CC family chemokine that specifically binds to CC chemokine receptor 7 (CCR7) [20]. CCR7 plays a role in the recirculation of normal lymphocytes and the homing of immune cells to the lymph nodes and spleen [21]. However, given the disseminating nature of leukemia cells, the mechanism by which different hematopoietic organs or tissues and their distinct microenvironmental cues impact the development of leukemia is largely unknown.

The BM is a major site for hematopoiesis in adult humans, whereas both the BM and spleen are important for hematopoiesis in mice [22,23]. Both the BM and spleen are involved in the development of leukemia, and other organs are also infiltrated by leukemia cells in the later stages [24]. There is a high incidence of splenomegaly in acute lymphoblastic leukemia (ALL), especially in acute T lymphoblastic leukemia (T-ALL), and splenomegaly has been observed to be related to the poor prognosis of leukemia patients [24,25]. However, the mechanisms underlying this association are not clear. In this study, we used the Notch1-induced mouse T-ALL model to compare the effects of the BM and spleen environments on a number of biological features, such as homing, proliferation and migration, of disseminated leukemia cells. We found that the spleen more potently recruited T-ALL cells by expressing a high level of MIP-3β. Furthermore, spleen cells promoted the proliferation and malignant phenotype of T-ALL cells, and splenectomy prolonged the survival time of leukemic mice. Thus, we provide the first systemic analysis of an organ-specific effect on the development of T-ALL cells and its underlying mechanisms.

## Results

### T-ALL cells disseminate differentially in hematopoietic organs

**Figure 1:**
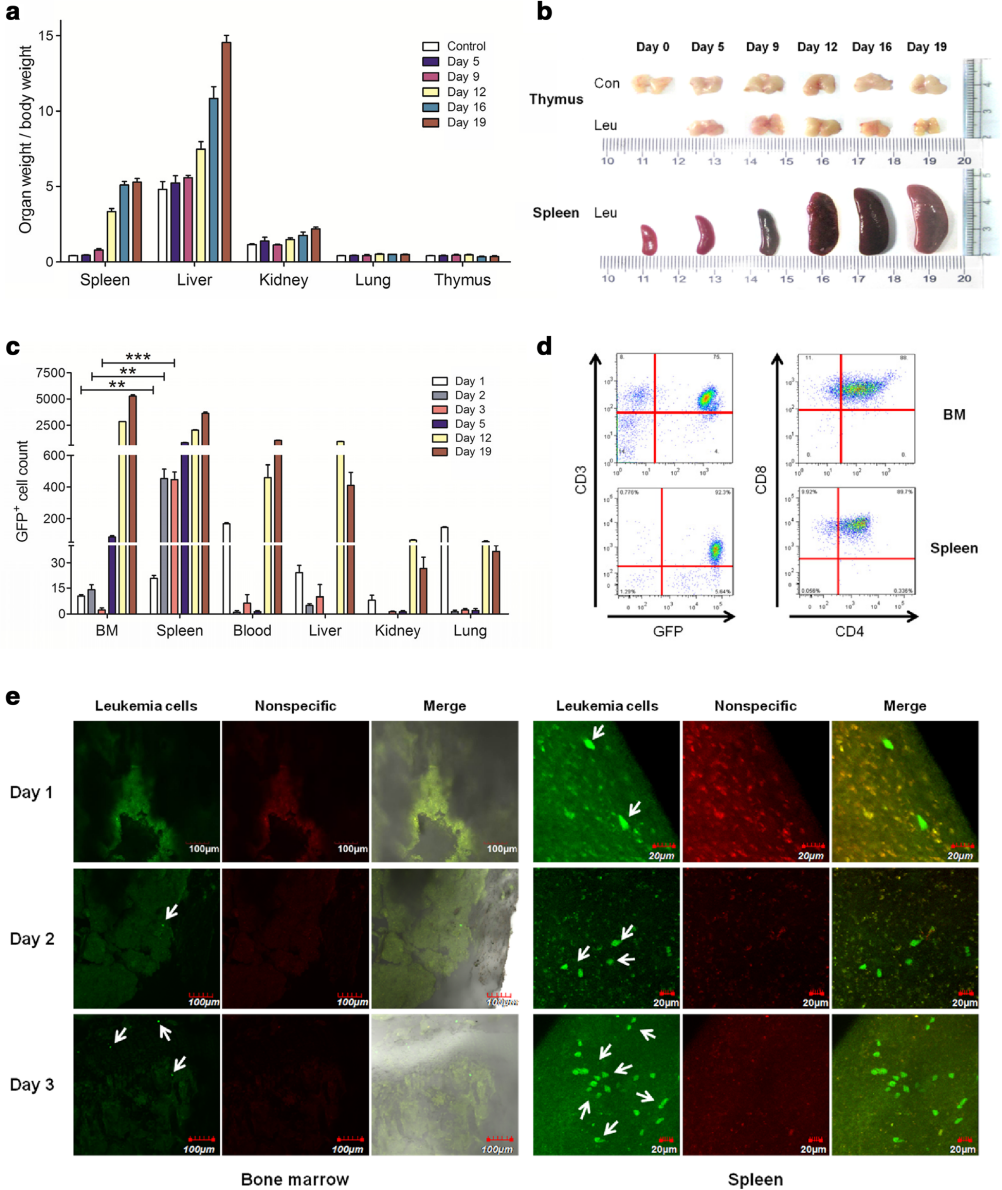
**Distribution of leukemia cells in different organs in the Notch1-induced mouse T-ALL model.** After injection of T-ALL cells, the size and weight of different organs and the population of T-ALL cells in these organs were measured at the indicated time points. **(a)** The weight of different organs was measured and calibrated by the respective body weight. **(b)** The typical sizes of the thymus and spleen are shown. **(c)** The population of T-ALL cells was determined by flow cytometry analysis and shown as cell counts per 10^4^ cells. **(d)** The phenotype of T-ALL cells was determined by flow cytometry analysis. **(e)** Two-photon fluorescence microscopy was used to track the distribution of leukemia cells in mice femurs (left) and spleen (right) within three days. GFP^+^ T-ALL cells are indicated by arrows. All the data are plotted as the means ± SD, and the statistical analysis was performed using Student’s *t*-test (**, p < 0.01; ***, p < 0.001).

Although leukemia cells will become dominant in the competition with normal hematopoietic cells, a suitable microenvironment is important for their establishment and rapid dissemination, especially during the initiation and early stages of leukemia. To better understand the impact of normal organ environments on leukemia cells, the Notch1-induced mouse T-ALL model [26] was modified in non-irradiated recipients. On day 9, a significant increase in absolute weight (organ weight/mouse weight) was only detected in the spleen. A final 12.25 ± 0.6802-fold increase was observed in the spleen (Figure 1a). The typical size of the spleen and thymus is illustrated in Figure 1b. The distribution of T-ALL cells in the different organs was analyzed by flow cytometry. Although an obvious increase in the number of T-ALL cells was detected in all organs analyzed at the late stage, the highest increase was observed in the BM and spleen but liver in early stage. Notably, the number of GFP^+^ cells in 10^4^ cells was significantly higher in the spleen than in the BM in the first five days after the injection of leukemia cells (p = 0.0063, 0.0021 and 0.0008, respectively) (Figure 1c). A phenotypic analysis showed that T-ALL cells from both the BM and spleen were CD3^+^CD4^+^CD8^+^ (Figure 1d).

To further confirm that the spleen provides a more suitable environment for the early development of Notch1-induced leukemia, the localization of T-ALL cells in the BM, spleen, thymus and liver was monitored by two-photon fluorescence microscopy. Fewer GFP^+^ cells were detected in the BM, whereas more T-ALL cells could be found in the spleen within the first 3 days (Figure 1e). Furthermore, unlike in spleen, T-ALL cells were undetectable in either thymus or liver in the first day (Additional file 1: Figure S1). Together, these data indicate that the spleen is more attractive for the residence of the disseminated T-ALL cells than other hematopoietic organs. We thus hypothesized that the spleen environment may recruit T-ALL cells and provide appropriate niches to support the survival, proliferation and dissemination of T-ALL cells.

### The spleen microenvironment preferentially recruits T-ALL cells

**Figure 2:**
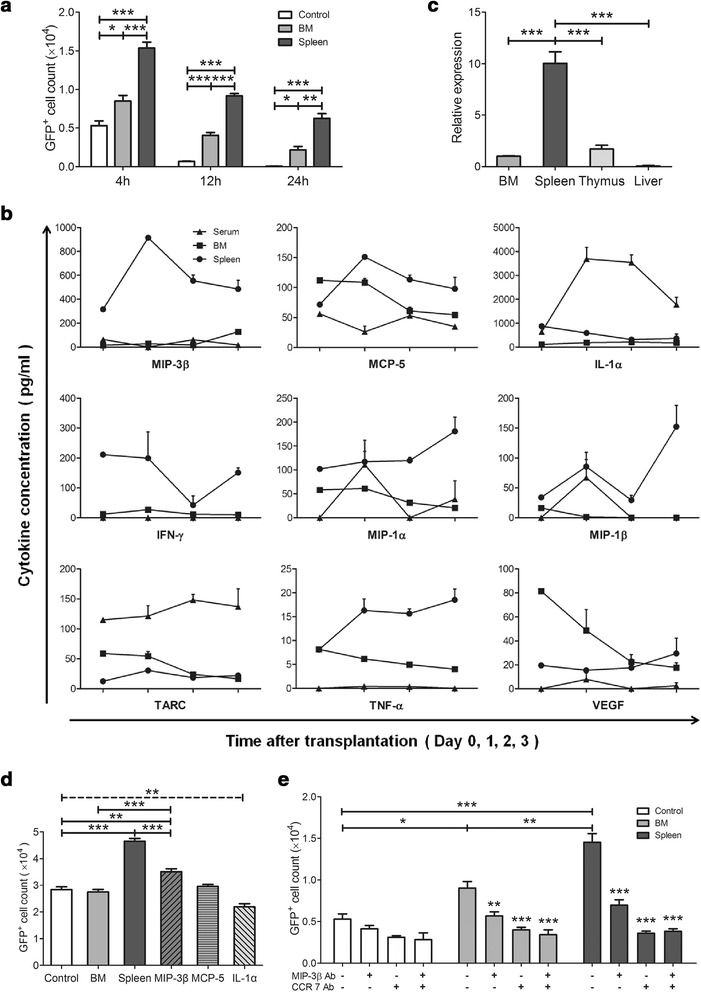
**Recruitment of T-ALL cells by the spleen environment. (a)** Single-cell suspension from the spleen or BM of normal mice was placed in the lower compartment of a transwell plate, and GFP^+^ cells were placed in the upper compartment. The GFP^+^ cells that migrated to the lower compartment were counted at the indicated time point using FACS analysis (n = 5). **(b)** The concentration (pg/ml) of cytokines/chemokines in BM, spleen and serum samples was determined by the MILLIPLEX® MAP Multiplex Immunoassay Kits. **(c)** Expression of MIP-3β in BM, spleen, thymus and liver cells from normal mice was measured by real-time PCR. **(d)** T-ALL cells were placed in the upper chamber of a transwell plate, and conditioned medium from normal spleen or BM cells, α-MEM medium or medium containing MIP-3β, MCP-5 or IL-1a was placed in the lower compartment. The GFP^+^ cells that migrated to the lower compartment in 4 hours were counted by FACS analysis (n = 5). **(e)** T-ALL cells were placed in the upper chambers, and α-MEM medium (Control), normal BM cells (BM) or spleen (Spleen) cells were placed in the lower chambers. Neutralizing antibodies against CCR7 and MIP-3β were in upper or lower chambers, respectively. The migrated GFP^+^ cells were counted at 4 hours by FACS (n = 5). All columns represent the means ± SD, and the statistical analysis was performed using one-way ANOVA with multiple comparison test (*, p < 0.05; **, p < 0.01; ***, p < 0.001).

To determine whether the spleen environment more potently recruits T-ALL cells, an *in vitro* cell migration assay was conducted using a transwell system. Significantly more GFP^+^ cells migrated to the lower compartments containing normal spleen cells than to those containing normal BM cells (Figure 2a). This observation suggests that the spleen environment more potently recruits T-ALL cells than the BM environment because of the higher level of soluble chemokines or cytokines expressed by spleen cells.

To further determine which chemokine or cytokine is important for this process, the concentration of a panel of cytokines/chemokines in the BM, spleen and peripheral blood samples was analyzed using MILLIPLEX® MAP Multiplex Immunoassay Kits. As shown in Figure 2b, the kinetics of the different chemokines/cytokines varied at the early stage. Notably, the physiological concentration of MIP-3β was higher in spleen samples than in BM or serum samples. Furthermore, the concentration of MIP-3β increased rapidly at day 1 and remained at a much higher level for three days. Real-time PCR analysis revealed that the spleen cells physiologically expressed a higher level of MIP-3β than BM, thymus or liver cells (Figure 2c).

It has been reported that activation of the Notch1 signaling pathway promotes the expression of CCR7 [27]. Therefore, an *in vitro* transwell experiment was performed to test the effect of MIP-3β; the addition of MIP-3β to the culture media in the lower compartment promoted the migration of T-ALL cells, although the magnitude of this effect was not as large as that of the spleen cells in the lower compartment (Figure 2d). To better confirm the effect of MIP-3β-CCR7 pathway, neutralizing antibodies were used in the transwell experiments. Addition of antibodies against either MIP-3β or CCR7 inhibited the migration of T-ALL cells (Figure 2e).

These results suggest that a high level of MIP-3β promotes the recruitment of T-ALL cells to the spleen in the early stages of T-ALL dissemination.

### The spleen environment further potentiates the malignance of T-ALL cells

**Figure 3:**
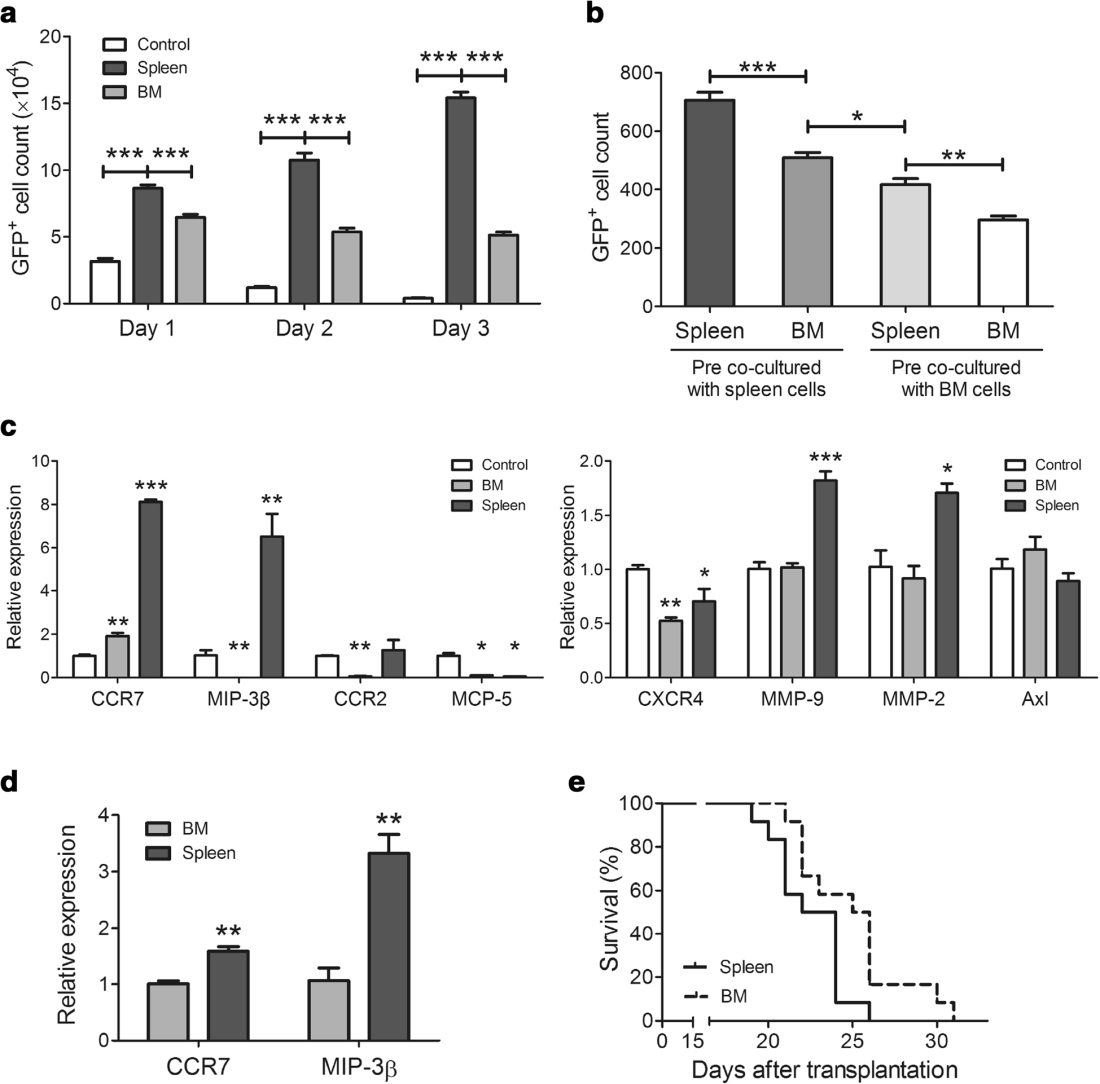
**Effects of spleen environment on the proliferation and migration of T-ALL cells. (a)** T-ALL cells were cultured with normal spleen or BM cells *in vitro* for the indicated periods, and the GFP^+^ cells were counted by FACS analysis (n = 5). **(b)** T-ALL cells were cultured with spleen or BM cells for 24 hours and then isolated and seeded into the upper compartment of a transwell plate, whereas the CM from spleen or BM cells was placed in the lower compartment. The GFP^+^ cells that migrated into the lower compartment after 24 hours were counted by FACS (n = 5). **(c)** The expression of migration-related genes in T-ALL cells was measured using real-time PCR after the T-ALL cells were co-cultured with spleen or BM cells for 24 hours. **(d)** 1 × 10^8^ T-ALL cells were transplanted into non-irradiated mice for 24 hours. The GFP^+^ cells were isolated from the BM or spleen and the expression of CCR7 and MIP-3β was analyzed by real-time PCR. **(e)** T-ALL cells were isolated from the BM of splenectomized leukemic mice or the spleen of sham-splenectomized leukemic mice, and an identical number of T-ALL cells was transplanted into two mouse groups (n = 12). The survival curves of the two groups are shown; splenectomy-BM-transplanted and sham-spleen-transplanted groups had a median survival time of 23 and 25.5 days, respectively (p < 0.05). All columns indicate the means ± SD, and the statistical analysis was performed using one-way ANOVA with multiple comparison test, Student’s *t* test and Kaplan-Meier survival analysis (*, p < 0.05; **, p < 0.01; ***, p < 0.001).

After studying the mechanism of how the spleen environment potently recruits T-ALL cells, we then evaluated whether the spleen environment affects T-ALL cells differently as opposed to BM. An *in vitro* co-culture assay was performed to examine the effects of normal BM or spleen cells on the proliferation of T-ALL cells and showed that spleen cells were more potent than BM cells for stimulating the proliferation of T-ALL cells (Figure 3a). The migration ability was analyzed after T-ALL cells were pre-co-cultured with BM or spleen cells using a transwell experiment. T-ALL cells pre-co-cultured with spleen cells migrated more efficiently to the lower chambers containing conditioned medium from either normal spleen cells or BM cells than those pre-co-cultured with BM cells (Figure 3b).

The expression of migration-related genes in T-ALL cells was also analyzed after they were co-cultured with spleen or BM cells. Significantly increased expression of MIP-3β, CCR7, MMP9 and MMP2 was observed when T-ALL cells were co-cultured with spleen cells but BM cells for 24 hours. However, little difference or decrease was observed in the expression of CCR2, MCP-5, CXCR4 and AX1 (Figure 3c). To further confirm this phenomenon *in vivo*, GFP^+^ cells were isolated from the BM or spleen 24 hours after injection of 1 × 10^8^ T-ALL cells, and expression of MIP-3β and CCR7 was analyzed by real-time PCR. As shown in Figure 3d, T-ALL cells isolated from the spleen expressed a higher level of both MIP-3β and CCR7.

The above results suggested that T-ALL cells in the spleen environment were more potent than those in the BM environment. To further verify this finding, an *in vivo* experiment was performed to compare the survival curves of two mouse groups that were transplanted with same number of GFP^+^ cells isolated from either the spleen or BM. To avoid a possible influence of the spleen on BM-residing leukemia cells, a splenectomized mouse T-ALL model was first established, when T-ALL cells were transplanted into mice after splenectomy. Then, GFP^+^ cells were isolated from the BM of the splenectomized leukemia mice. For the spleen-resident T-ALL cells, a sham-operated leukemia model was established first, and GFP^+^ cells were isolated from the spleen of the sham-operated leukemia mice. The spleen-resident T-ALL cells had a shorter life span than BM-resident leukemia cells (p = 0.0299, Figure 3e).

### Splenectomy prolongs the survival of T-ALL-bearing mice

**Figure 4:**
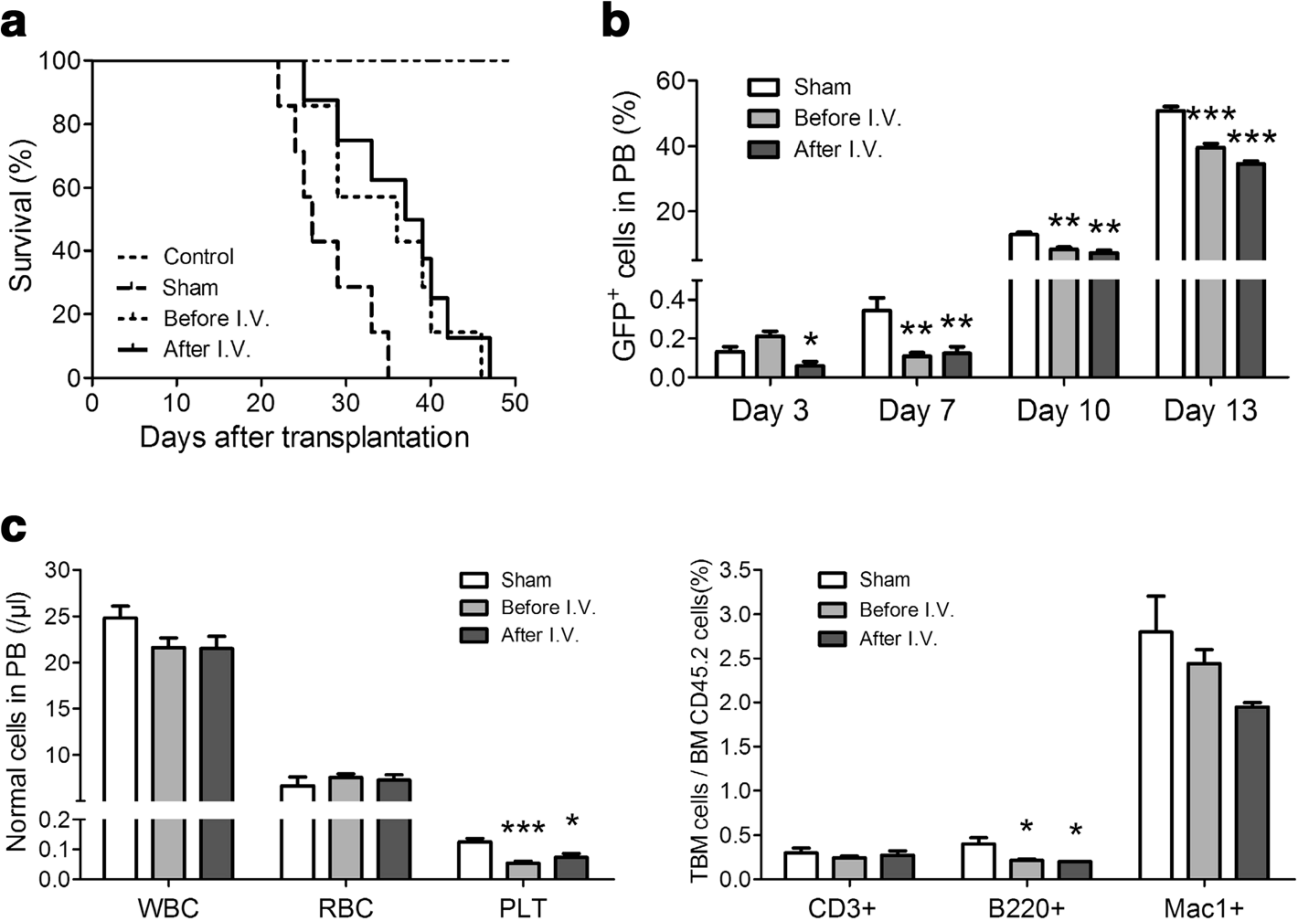
**Impact of splenectomy on the survival of T-ALL mice.** Mice were splenectomized without injection of T-ALL cells in the control group, a sham operation was conducted 24 hours after injection in the sham group, mice were splenectomized 1 week before injection in the before-I.V. group. The mice were splenectomized 24 hours after injection in the after-I.V. group. **(a)** Survival curves of the different mouse groups (n ≥ 7). The median survival time of the two groups undergoing splenectomy (36 and 38 days) was significantly longer than that of the sham group (26 days, p < 0.05). **(b)** The percentage of GFP^+^ leukemia cells in the peripheral blood was determined by FACS at the indicated time points. **(c)** Left: comparison of the normal peripheral blood cells in three leukemic mouse groups on day 23 (1 × 10^3^ for WBC and 1 × 10^6^ for RBC and Plts). Right: the proportion of normal BM CD3^+^, B220^+^ and Mac1^+^ cells in the three leukemic mouse groups on day 23. All columns represent the means ± SDs, and the statistical analysis was performed using Kaplan-Meier survival analysis and one-way ANOVA with multiple comparison test (*, P < 0.05; **, p < 0.01; ***, p < 0.001).

Because we have shown that the spleen is the main reservoir for early stage T-ALL cells and that the spleen can cultivate T-ALL cells to be more potent during the development of leukemia, it is of interest to determine whether removal of the spleen would be beneficial to the survival of leukemia mice. Splenectomy was conducted either before or after the transplantation of T-ALL cells. No death was found in the control group, in which the animals underwent splenectomy but did not receive injections of T-ALL cells. The survival time of the mice in the two splenectomy groups was similar and significantly longer than that of the sham group (Figure 4a).

Peripheral blood GFP^+^ leukemia cells were monitored by FACS. As shown in Figure 4b, the number of T-ALL cells was low in the early stage of the two groups that underwent splenectomy but increased steadily in the sham operation group. To exclude the potential influence of splenectomy on normal hematopoiesis, we also quantified the blood cells in each lineage. No difference was observed in the red blood cell (RBC) count, whereas a slight decrease in the white blood cell (WBC) and platelet counts was detected in the two groups undergoing splenectomy (Figure 4c left). The percentage of normal T cells (CD3^+^), B cells (B220^+^) and myeloid cells (Mac1^+^) in the BM from the three groups was also analyzed, and only slight decreases in B220^+^ and Mac1^+^ cells were observed (Figure 4c right). These results suggest that splenectomy had little influence on the maturation and differentiation of normal hematopoietic cells in a T-ALL environment and do not support the possibility that removal of the spleen enhances normal blood cell production.

## Discussion

The extracellular environment has great impacts on the development and relapse of malignancies. In recent years, the importance of the niche for leukemia cells and other malignancies has been proposed [28]. In leukemia, multiple organs are involved and infiltrated by leukemia cells at the later disease stages [23]. However, the importance of different organs for the development of leukemia is not fully understood. To study the initial localization of leukemia cells and the impacts of the specific environment on leukemia cells, a non-irradiated Notch1-induced mouse T-ALL model was applied. Analysis of this model showed that the most obviously enlarged organ was the spleen. The earliest existence and increase of T-ALL cells was observed in the spleen and not the BM, thymus or liver, and this result was confirmed by two-photon microscopy. An *in vitro* migration assay further confirmed that T-ALL cells preferentially migrate to the spleen environment. Therefore, the spleen attracts T-ALL cells and provides a suitable supportive microenvironment for the development of T-ALL.

The migration ability of T-ALL cells to various different organs may be associated with the chemokine profile of the organs and the receptor profile on the surface of T-ALL cells. Abnormal expression of chemokine receptors, such as CCR7 [29,30], CCR5 [31] and CCR9 [32], on Notch1-induced T-ALL cells has been observed. By screening the chemokine expression profiles in the spleen, BM and peripheral blood, we obtained the expression pattern of a panel of chemokines during the early stages of T-ALL. Notably, the expression of MIP-3β was higher in the spleen, and a sustained increase at the early stage was observed. Real-time PCR analysis further confirmed that spleen cells expressed a higher level of MIP-3β than BM cells. MIP-3β shares its receptor, CCR7, with CCL21, and abnormal activation of Notch1 may enhance the expression of CCR7 [27]. We found that leukemia cells expressed a basal level of CCR7, whereas expression of MIP-3β was undetectable, and co-culture of T-ALL cells with spleen cells increased the expression of both of these genes in leukemia cells. Furthermore, an *in vitro* transwell experiment confirmed that MIP-3β enhances the migration of T-ALL cells. The effects could be blocked by neutralizing antibodies against both MIP-3β and CCR7. Therefore, a high level of MIP-3β in the spleen environment may initially recruit T-ALL cells, and the presence of T-ALL cells in the spleen will further recruit T-ALL cells to the spleen by expressing additional MIP-3β. Therefore, we conclude that the MIP-3β/CCR7 pathway plays a central role in the localization of T-ALL cells to the spleen, especially during the early stage of T-ALL.

Previous studies suggested that chemokines and cytokines in the spleen microenvironment contribute to the development of leukemia. Monocyte chemo-attractant protein 5 (MCP-5), which is expressed constitutively in the thymus and lymph nodes, has been reported to contribute to accelerated overgrowth of F-MuLV-induced erythroleukemic cells [33]. In CML, SDF-1/CXCL12, an important component of the HSC niche [34], contributes to increased relocalization of long-term HSCs to the spleen at later time points [35]. In our study, the basal level of MCP-5 expression was not higher in the spleen than in the BM, and only a slight increase was observed in the early stage of T-ALL. Furthermore, expression of the MCP-5 receptor, CCR2, in T-ALL cells was low, and no increase was observed when T-ALL cells were co-cultured with spleen cells (data not shown). Most importantly, MCP-5 did not chemoattract T-ALL cells in the *in vitro* migration assay. Additionally, expression of CXCL12 was not high in the spleen (data not shown) in the early stage of T-ALL, and expression of CXCR4 was unchanged when T-ALL cells were co-cultured with spleen cells or BM cells. These results suggest that different mechanisms exist in different leukemia models.

The malignant behavior of tumor cells is governed by intrinsic lesions and affected by the surrounding microenvironment [3,36,37]. Different mechanisms for this effect have been proposed [38,39]. MIP-3β is mainly expressed in secondary lymphoid organs and plays important roles in directing the migration of naïve T cells, B cells and dendritic cells to sites of antigen presentation [40,41]. CCR7 is regarded as an important lymphocyte “homing receptor” [29]. Over expression of these factors occurs in tumors. Expression of CCR7 in breast cancer, melanoma and other malignant cells was found to be associated with tumor growth, angiogenesis, invasion and lymph node metastasis [42-44]. The involvement of this pathway was also studied in CLL and B-cell acute lymphocytic leukemia (B-ALL), and MIP-3β was reported to enhance the apoptotic resistance of B-ALL [45,46]. Furthermore, expression of murine CCR7 in CCR7- human T-ALL cells enables these cells to infiltrate into the mouse central nervous system [30]. We observed that co-culture with spleen cells stimulated the proliferation of T-ALL cells. Furthermore, the splenic microenvironment up-regulated both CCR7 and MIP-3β in T-ALL cells. Leukemic mice injected with spleen-resident T-ALL cells had a shorter lifespan than those injected with BM-resident T-ALL cells from spleen-free leukemia mice. These observations suggest that T-ALL cells in the spleen environment are more potent, partly because of increased expression of CCR7 and MIP-3β.

Because the spleen is important for development of T-ALL, we hypothesized that splenectomy may be beneficial for leukemia mice. Removal of the spleen, either before or after the injection of leukemia cells, prolonged the survival of leukemic mice. Prolonged survival of leukemic mice that received splenectomy has also been observed in other leukemia models [33]. However, removal of the spleen did not abolish the development of T-ALL, which suggests that the spleen provides a more favorable environment, but not an indispensable factor, for the survival and dissemination of T-ALL cells. The clinical relevance between the severity of spleen involvement and the prognosis of leukemia patients has long been observed [24]. In particular, the spleen is considered to be a critical organ for cancer progression and metastasis [47]. However, our current data do not demonstrate whether spleen has critical roles in the transformation of normal hematopoietic cells into T-ALL cells but instead indicate its important effects on the dissemination of T-ALL cells. In addition, the specific role of spleen on T-ALL may not be generalized since splenectomy had little effect on the survival of the leukemia mice induced by MLL-AF9 (data not shown). Nevertheless, the increased migration of the T-ALL leukemia cells to the spleen is owing to enhanced chemotaxis of T-ALL cells to spleen via the MIP-3β-CCR7 pathway, not just normal homing phenomenon. Moreover, the splenic microenvironment further promotes the malignancies of T-ALL cells.

**Figure 5:**
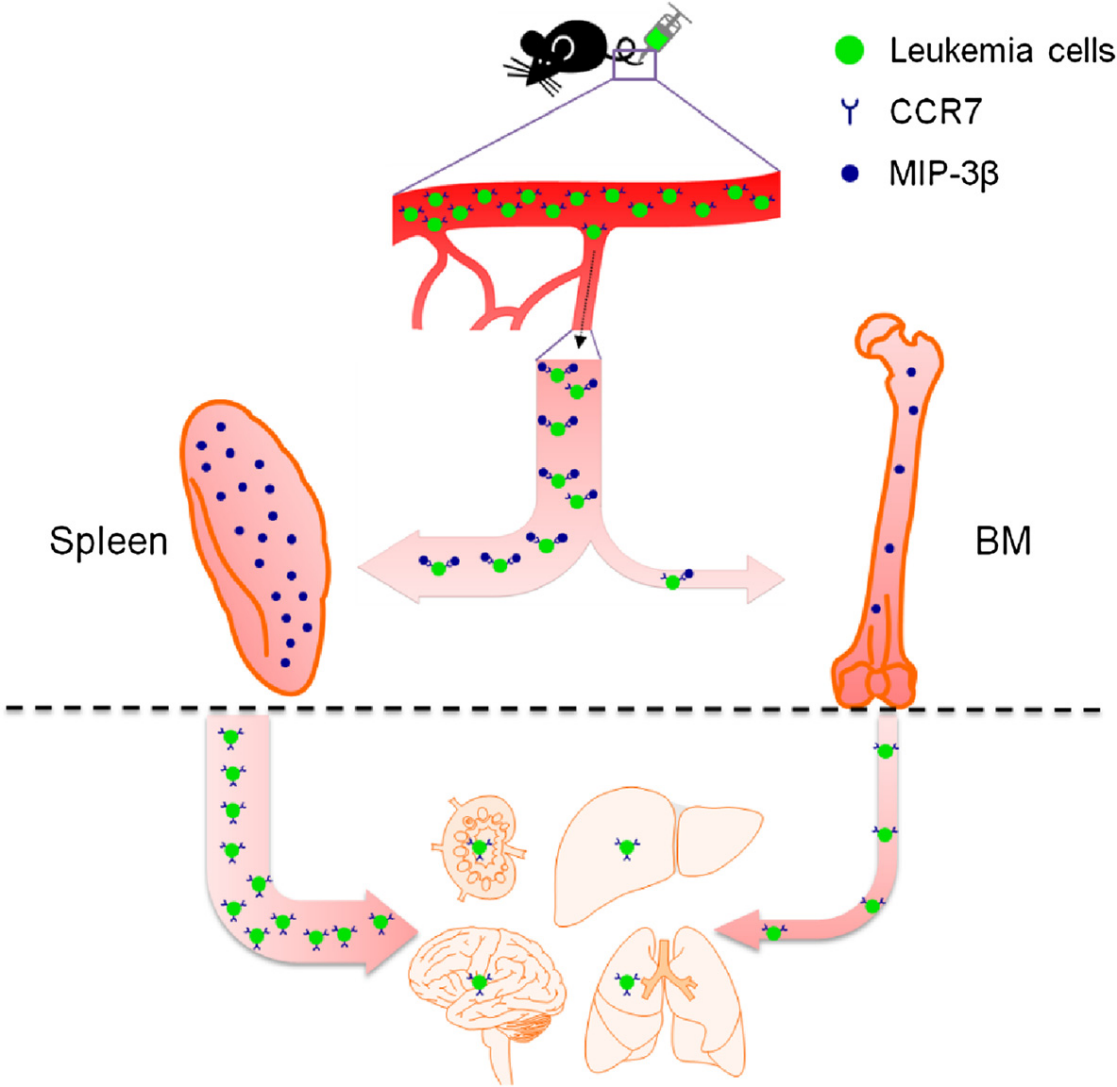
**A proposed model for the promoting effect of splenic microenvironment on disseminated T-ALL cells.** The splenic microenvironment has high level of MIP-3β, which recruits T-ALL cells expressing a high level of CCR7. The splenic microenvironment stimulates T-ALL cells to express a higher level of MIP-3β, which further recruits T-ALL cells to the spleen. The splenic microenvironment promotes the proliferation of T-ALL cells, stimulates the expression of CCR7 and enhances the migration ability of T-ALL cells.

In summary, our current study demonstrates an organ specific effect on T-ALL development. The splenic microenvironmental cues promote the development of T-ALL. MIP-3β, which is highly expressed in the splenic microenvironment, recruits Notch1-induced T-ALL cells, which express a high level of CCR7. The splenic environment stimulates T-ALL cells to express a higher level of MIP-3β, which further recruits T-ALL cells to the spleen. Moreover, the splenic environment promotes the proliferation of T-ALL cells, stimulates the expression of CCR7 and enhances the migration ability of T-ALL cells (Figure 5), thus promoting their malignancy, and removal of spleen can prolong the survival of leukemic mice. Therefore, spleen microenvironment may serve as a therapeutic target for the treatment of some types of leukemia.

## Methods

### Mice and antibodies

C57BL/6 J (CD45.2) and B6.SJL-PtprcaPepcb/BoyJ mice (B6.SJL, CD45.1) were provided by the Animal Centre of the Institute of Hematology & Blood Diseases Hospital, CAMS & PUMC. Sex- and age-matched mice were used and maintained in the SPF-certified animal facility in the same center. The procedures for the animal experiments were approved by the Animal Care and Use Committee at the institutions involved in this study. After transplantation, the mice were raised in sterile squirrel cages in a positive pressure room. All of the antibodies used in this paper were obtained from eBioscience (San Diego, CA, USA), including PE-, FITC-, or PerCP-Cy5.5-conjugated anti-CD45.1, PE-conjugated anti-CD45.2, PE-conjugated anti-Sca-1, APC-conjugated anti-c-Kit, PE-conjugated anti-CD3, PE-Cy7-conjugated anti-B220, APC-conjugated anti-Mac-1, biotin-conjugated lineage markers (anti-CD3, −CD4, −CD8, −B220, −CD11b, −Gr-1, and -Ter119) and streptavidin-PE-Cy7. Neutralizing antibodies against MIP-3β and CCR7 were purchased from R&D Systems (Minneapolis, MN, USA).

### Notch1 induced T-ALL mouse model

The establishment of the Notch1-induced mouse T-ALL model was described previously [17,26,48]. To mimic the development of leukemia, a non-irradiated T-ALL model was developed in this study. Briefly, GFP^+^CD45.1^+^ leukemia cells were isolated from either the spleen or bone marrow of B6.SJL leukemia mice and transplanted into C57BL/6 J female mice (1 × 10^6^ cells/mouse) without irradiation. The survival time of the mice was recorded (the day that the animals underwent transplantation was designated as day 0), and the mice were sacrificed at the time points indicated. Different organs, including the liver, spleen, lymph nodes, lung, kidney, thymus and BM, were collected for further analysis.

### Flow cytometric analysis and cell sorting

Standard protocols were followed for all experiments. An LSR II flow cytometer (BD Biosciences) was used for FACS analysis, and a FACS Aria II or Influx (BD Biosciences) was used for cell sorting.

### Examination of T-ALL cells by two-photon microscopy

The non-irradiated T-ALL model was established and mice were sacrificed 1, 2 or 3 days post-transplantation. The localization of T-ALL cells in BM, spleen, thymus and liver was determined using two-photon microscopy (FV1000, Olympus Corporation) after cardiac perfusion to remove T-ALL cells in blood vessels and sinus following the protocol described previously [49] with some modifications. For spleen sample preparation, the whole spleen was fixed in 4% PFA/PBS at 4°C for 10 hours and then cryo-protected in 20% sucrose/PBS at 4°C for 24 hours. The spleen samples were re-fixed with 4% PFA/PBS for 20 min at room temperature and then incubated in SCALEVIEW-A2 (Olympus Corporation) for 30 days. Finally, the spleen samples were cut vertically and fixed in a 0.5% agarose gel before two-photon analysis. At least 3 independent mouse samples were analyzed.

### Splenectomy

To prepare the animals for splenectomy, the upper left side abdominal skin of C57BL/6 J female mice was cleaned by Na_2_S (0.8%, m/v) 1 day before operation. Mice were anaesthetized with ether, and the splenic artery and vein were triple ligated before removal of the spleen. The muscle, peritoneum and skin were closed in separate layers via sterile 4–0 silk braided non-absorbable sutures. The drinking water for the animals after the operation was supplemented with Enrofloxacin solution (Sichuan China, Bayer). In the sham splenectomy group, the identical operation was conducted but without the splenectomy. All of the mice in the control splenectomy group survived the surgery and were sacrificed after 6 months.

### Real-time RT-PCR analysis

Total RNA was extracted with an RNeasy mini kit (Qiagen) or Trizol (Invitrogen) according to the manufacturer’s instructions. Reverse transcription was achieved using a QuantiTect Reverse Transcription Kit (Qiagen). Real-time PCR was performed using an ABI-Prism 7500 Sequence Detector (Applied Biosystems, Foster City, CA). The sequences of all primers are listed in Additional file 1: Table S1.

### In vitro cell migration assay

The migration of T-ALL cells was analyzed using Falcon 8.0 pore size PET track-etched membrane following the manufacturer’s instructions. Freshly isolated GFP^+^ cells from BM were seeded at 1 × 10^5^ cells/well in 200 μl α-MEM medium supplemented with 15% fetal bovine serum (FBS) in the upper chamber. In some experiments, BM or spleen cells were cultured in the lower chambers in 500 μl same medium at 1 × 10^6^ cells/well for 24 hours before migration assay. To study the different potential of the BM or spleen environment to attract leukemia cells, whole spleen cells or BM cells from normal mice were in the lower chambers. To study the effects of candidate cytokines, the lower chambers were filled with medium alone (negative control), conditioned medium from normal spleen or BM cells, or medium supplemented with 100 ng/ml MIP-3β, MCP-5 or IL-1a, respectively. In neutralizing experiment, two antibodies were used alone or in combination at the final concentration of 2 μg/ml each and pretreated for 30 min at 37°C before migration assay. Antibody against MIP-3β was added to the lower chambers and T-ALL cells were preincubated with antibody against CCR7 before being transferred to upper chambers. To study the impact of normal cells on the migration ability of T-ALL cells, co-culture was first performed using Nunc 0.3 pore size PET track-etched membrane. T-ALL cells were cultured in the upper chambers, and normal BM or spleen cells were cultured in the lower chambers for the indicated times. The T-ALL cells were then harvested and transferred to the upper chambers (8.0 pore size), while the lower chambers contained control medium or the conditioned medium of normal spleen cells or BM cells, respectively. At the end of each experiment, the upper chambers were removed and the cells in the lower chambers were resuspended and collected thoroughly. Total cells in the lower chambers were counted by flow cytometry and T-ALL cells migrated into the lower chamber were counted by gating the GFP^+^ cells.

### Co-culture assay

Freshly isolated GFP^+^ cells and spleen or BM cells were seeded into 24-well plates at 5 × 10^4^ cells/well and 5 × 10^5^ cells/well, respectively. The cells were then co-cultured for the indicated times, and the GFP^+^ cells were counted by flow cytometry.

### Cytokine/Chemokine analysis by immunoassays

The non-irradiated T-ALL model was established by injecting 1 × 10^6^ T-ALL cells/mouse, and the leukemic mice were sacrificed at different time points. The analysis of cytokine/chemokine was followed the protocol described previously [35] with slight modification. Peripheral blood was obtained from the eye vein, and serum samples were collected following a standard protocol. For BM samples, bilateral femurs and fibulae were intramedullarily flushed with 500 μl of phosphate-buffered saline (PBS), and the supernatant was collected by centrifugation. For spleen samples, each spleen was ground in 500 μl of PBS, and the filter was rinsed with an extra 500 μl of PBS. The supernatant was then collected by centrifugation. The concentration of cytokines/chemokines (mouse IL-1α, IFN-g, MIP-1α, MIP-1β, RANTES, TNF-α, VEGF, MCP-5, MIP-3β, and TRAC) in these samples was detected by the MILLIPLEX® MAP Multiplex Immunoassay Kits (Mouse Cytokine/Chemokine Panel I, MCYTOMAG-70 K and Mouse Cytokine/Chemokine Panel II, MCYP2 MAG-73 K; Merck Millipore) following the manufacturer’s instructions. At least 3 independent mouse samples were analyzed.

### Statistical analysis

The data are plotted as the means ± SD. Comparisons between two groups were analyzed by Student’s *t* test, whereas comparisons among more than three groups were analyzed by one-way ANOVA with multiple comparison tests. Survival analysis was performed using Kaplan-Meier statistics (*p < 0.05; **p ≤ 0.01; ***p ≤ 0.001).

## Additional file

Additional file 1: Table S1. Primers used for real-time PCR. **Figure S1.** The distribution of leukemia cells in mice thymus (top) and liver (bottom) was observed using two-photon fluorescence microscopy the first day after transplantation.

## References

[1] DuFort CC, Paszek MJ, Weaver VM (2011). Balancing forces: architectural control of mechanotransduction. Nat Rev Mol Cell Biol.

[2] Chang AH, Parsonnet J (2010). Role of bacteria in oncogenesis. Clin Microbiol Rev.

[3] Lewis CE, Pollard JW (2006). Distinct role of macrophages in different tumor microenvironments. Cancer Res.

[4] Hu B, Castillo E, Harewood L, Ostano P, Reymond A, Dummer R, Raffoul W, Hoetzenecker W, Hofbauer GF, Dotto GP (2012). Multifocal epithelial tumors and field cancerization from loss of mesenchymal CSL signaling. Cell.

[5] Raaijmakers MH, Mukherjee S, Guo S, Zhang S, Kobayashi T, Schoonmaker JA, Ebert BL, Al-Shahrour F, Hasserjian RP, Scadden EO, Aung Z, Matza M, Merkenschlager M, Lin C, Rommens JM, Scadden DT (2010). Bone progenitor dysfunction induces myelodysplasia and secondary leukaemia. Nature.

[6] Colmone A, Amorim M, Pontier AL, Wang S, Jablonski E, Sipkins DA (2008). Leukemic cells create bone marrow niches that disrupt the behavior of normal hematopoietic progenitor cells. Science.

[7] Cairns RA, Harris IS, Mak TW (2011). Regulation of cancer cell metabolism. Nat Rev Cancer.

[8] Kiel MJ, Morrison SJ (2008). Uncertainty in the niches that maintain haematopoietic stem cells. Nat Rev Immunol.

[9] Mercier FE, Ragu C, Scadden DT (2012). The bone marrow at the crossroads of blood and immunity. Nat Rev Immunol.

[10] Ehninger A, Trumpp A (2011). The bone marrow stem cell niche grows up: mesenchymal stem cells and macrophages move in. J Exp Med.

[11] Ramakrishnan A, Deeg HJ (2009). A novel role for the marrow microenvironment in initiating and sustaining hematopoietic disease. Expert Opin Biol Ther.

[12] Liu N, Zhang J, Ji C: **The emerging roles of Notch signaling in leukemia and stem cells.** *Biomark Res* 2013, **1:**23. 10.1186/2050-7771-1-23 PMC4177577 24252593

[13] Zou J, Li P, Lu F, Liu N, Dai J, Ye J, Qu X, Sun X, Ma D, Park J, Ji C: **Notch1 is required for hypoxia-induced proliferation, invasion and chemoresistance of T-cell acute lymphoblastic leukemia cells.** *J Hematol Oncol* 2013, **6:**3. 10.1186/1756-8722-6-3 PMC3544631 23289374

[14] Lane SW, Scadden DT, Gilliland DG (2009). The leukemic stem cell niche: current concepts and therapeutic opportunities. Blood.

[15] Wang L, Cheng T, Zheng G (2013). The impact of tumor microenvironments on stem cells. Transl Cancer Res.

[16] Boyerinas B, Zafrir M, Yesilkanal AE, Price TT, Hyjek EM, Sipkins DA (2013). Adhesion to osteopontin in the bone marrow niche regulates lymphoblastic leukemia cell dormancy. Blood.

[17] Tian C, Zheng G, Cao Z, Li Q, Ju Z, Wang J, Yuan W, Cheng T (2013). Hes1 mediates the different responses of hematopoietic stem and progenitor cells to T cell leukemic environment. Cell Cycle.

[18] Allen SJ, Crown SE, Handel TM (2007). Chemokine: receptor structure, interactions, and antagonism. Annu Rev Immunol.

[19] Bachmann MF, Kopf M, Marsland BJ (2006). Chemokines: more than just road signs. Nat Rev Immunol.

[20] Forster R, Davalos-Misslitz AC, Rot A (2008). CCR7 and its ligands: balancing immunity and tolerance. Nat Rev Immunol.

[21] Srour EF, Jetmore A, Wolber FM, Plett PA, Abonour R, Yoder MC, Orschell-Traycoff CM (2001). Homing, cell cycle kinetics and fate of transplanted hematopoietic stem cells. Leukemia.

[22] Neill HC O (2012). Niches for Extramedullary Hematopoiesis in the Spleen. Niche J.

[23] Kaushansky K, Lichtman MA, Beutler E, Kipps TJ, Seligsohn U, Prchal JT (2010). Williams hematology.

[24] Shuster JJ, Falletta JM, Pullen DJ, Crist WM, Humphrey GB, Dowell BL, Wharam MD, Borowitz M (1990). Prognostic factors in childhood T-cell acute lymphoblastic leukemia: a Pediatric Oncology Group study. Blood.

[25] Kienle DL (2013). The spleen in hematologic malignancies. Ther Umsch.

[26] Hu X, Shen H, Tian C, Yu H, Zheng G, XuFeng R, Ju Z, Xu J, Wang J, Cheng T (2009). Kinetics of normal hematopoietic stem and progenitor cells in a Notch1-induced leukemia model. Blood.

[27] Koch U, Radtke F (2007). Notch and cancer: a double-edged sword. Cell Mol Life Sci.

[28] Borovski T, De Sousa EMF, Vermeulen L, Medema JP (2011). Cancer stem cell niche: the place to be. Cancer Res.

[29] Campbell JJ, Bowman EP, Murphy K, Youngman KR, Siani MA, Thompson DA, Wu L, Zlotnik A, Butcher EC (1998). 6-C-kine (SLC), a lymphocyte adhesion-triggering chemokine expressed by high endothelium, is an agonist for the MIP-3beta receptor CCR7. J Cell Biol.

[30] Buonamici S, Trimarchi T, Ruocco MG, Reavie L, Cathelin S, Mar BG, Klinakis A, Lukyanov Y, Tseng JC, Sen F, Gehrie E, Li M, Newcomb E, Zavadil J, Meruelo D, Lipp M, Ibrahim S, Efstratiadis A, Zagzag D, Bromberg JS, Dustin ML, Aifantis I (2009). CCR7 signalling as an essential regulator of CNS infiltration in T-cell leukaemia. Nature.

[31] Mirandola L, Chiriva-Internati M, Montagna D, Locatelli F, Zecca M, Ranzani M, Basile A, Locati M, Cobos E, Kast WM, Asselta R, Paraboschi EM, Comi P, Chiaramonte R (2012). Notch1 regulates chemotaxis and proliferation by controlling the CC-chemokine receptors 5 and 9 in T cell acute lymphoblastic leukaemia. J Pathol.

[32] Koch U, Radtke F (2011). Mechanisms of T cell development and transformation. Annu Rev Cell Dev Biol.

[33] Shaked Y, Cervi D, Neuman M, Chen L, Klement G, Michaud CR, Haeri M, Pak BJ, Kerbel RS, Ben-David Y (2005). The splenic microenvironment is a source of proangiogenesis/inflammatory mediators accelerating the expansion of murine erythroleukemic cells. Blood.

[34] Sugiyama T, Kohara H, Noda M, Nagasawa T (2006). Maintenance of the hematopoietic stem cell pool by CXCL12-CXCR4 chemokine signaling in bone marrow stromal cell niches. Immunity.

[35] Zhang B, Ho YW, Huang Q, Maeda T, Lin A, Lee SU, Hair A, Holyoake TL, Huettner C, Bhatia R (2012). Altered microenvironmental regulation of leukemic and normal stem cells in chronic myelogenous leukemia. Cancer Cell.

[36] Welch JS, Ley TJ, Link DC, Miller CA, Larson DE, Koboldt DC, Wartman LD, Lamprecht TL, Liu F, Xia J, Kandoth C, Fulton RS, McLellan MD, Dooling DJ, Wallis JW, Chen K, Harris CC, Schmidt HK, Kalicki-Veizer JM, Lu C, Zhang Q, Lin L, O'Laughlin MD, McMichael JF, Delehaunty KD, Fulton LA, Magrini VJ, McGrath SD, Demeter RT, Vickery TL (2012). The origin and evolution of mutations in acute myeloid leukemia. Cell.

[37] Wang L, Zheng GG, Ma CH, Lin YM, Zhang HY, Ma YY, Chong JH, Wu KF (2008). A special linker between macrophage and hematopoietic malignant cells: membrane form of macrophage colony-stimulating factor. Cancer Res.

[38] Hanahan D, Weinberg RA (2011). Hallmarks of cancer: the next generation. Cell.

[39] Wels J, Kaplan RN, Rafii S, Lyden D (2008). Migratory neighbors and distant invaders: tumor-associated niche cells. Genes Dev.

[40] Payne D, Drinkwater S, Baretto R, Duddridge M, Browning MJ (2009). Expression of chemokine receptors CXCR4, CXCR5 and CCR7 on B and T lymphocytes from patients with primary antibody deficiency. Clin Exp Immunol.

[41] Kursar M, Hopken UE, Koch M, Kohler A, Lipp M, Kaufmann SH, Mittrucker HW (2005). Differential requirements for the chemokine receptor CCR7 in T cell activation during Listeria monocytogenes infection. J Exp Med.

[42] Shields JD, Fleury ME, Yong C, Tomei AA, Randolph GJ, Swartz MA (2007). Autologous chemotaxis as a mechanism of tumor cell homing to lymphatics via interstitial flow and autocrine CCR7 signaling. Cancer Cell.

[43] Zhang Q, Sun L, Yin L, Ming J, Zhang S, Luo W, Qiu X (2013). CCL19/CCR7 upregulates heparanase via specificity protein-1 (Sp1) to promote invasion of cell in lung cancer. Tumour Biol.

[44] Berndt B, Haverkampf S, Reith G, Keil S, Niggemann B, Zanker KS, Dittmar T: **Fusion of CCL21 non-migratory active breast epithelial and breast cancer cells give rise to CCL21 migratory active tumor hybrid cell lines.** *PLoS One* 2013, **8:**e63711. 10.1371/journal.pone.0063711 PMC3646822 23667660

[45] Catusse J, Leick M, Groch M, Clark DJ, Buchner MV, Zirlik K, Burger M: **Role of the atypical chemoattractant receptor CRAM in regulating CCL19 induced CCR7 responses in B-cell chronic lymphocytic leukemia.** *Mol Cancer* 2010, **9:**297. 10.1186/1476-4598-9-297 PMC2998479 21092185

[46] Corcione A, Arduino N, Ferretti E, Pistorio A, Spinelli M, Ottonello L, Dallegri F, Basso G, Pistoia V (2006). Chemokine receptor expression and function in childhood acute lymphoblastic leukemia of B-lineage. Leuk Res.

[47] McAllister SS, Weinberg RA (2014). The tumour-induced systemic environment as a critical regulator of cancer progression and metastasis. Nat Cell Biol.

[48] Grabher C, von Boehmer H, Look AT (2006). Notch 1 activation in the molecular pathogenesis of T-cell acute lymphoblastic leukaemia. Nat Rev Cancer.

[49] Xie Y, Yin T, Wiegraebe W, He XC, Miller D, Stark D, Perko K, Alexander R, Schwartz J, Grindley JC, Park J, Haug JS, Wunderlich JP, Li H, Zhang S, Johnson T, Feldman RA, Li L (2009). Detection of functional haematopoietic stem cell niche using real-time imaging. Nature.

