# Supplementary material for: Notch1-induced T cell leukemia can be potentiated by microenvironmental cues in the spleen
Source: J Hematol Oncol. 2014 Nov 4;7:71. doi: 10.1186/s13045-014-0071-7 (PMC4229605; doi:10.1186/s13045-014-0071-7)
Supplement: Additional file 1: Table S1. — Primers used for real-time PCR. Figure S1. The distribution of leukemia cells in mice thymus (top) and liver (bottom) was observed using two-photon fluorescence microscopy the first day after transplantation. [file 13045_2014_71_MOESM1_ESM.pptx]

## Slide 1
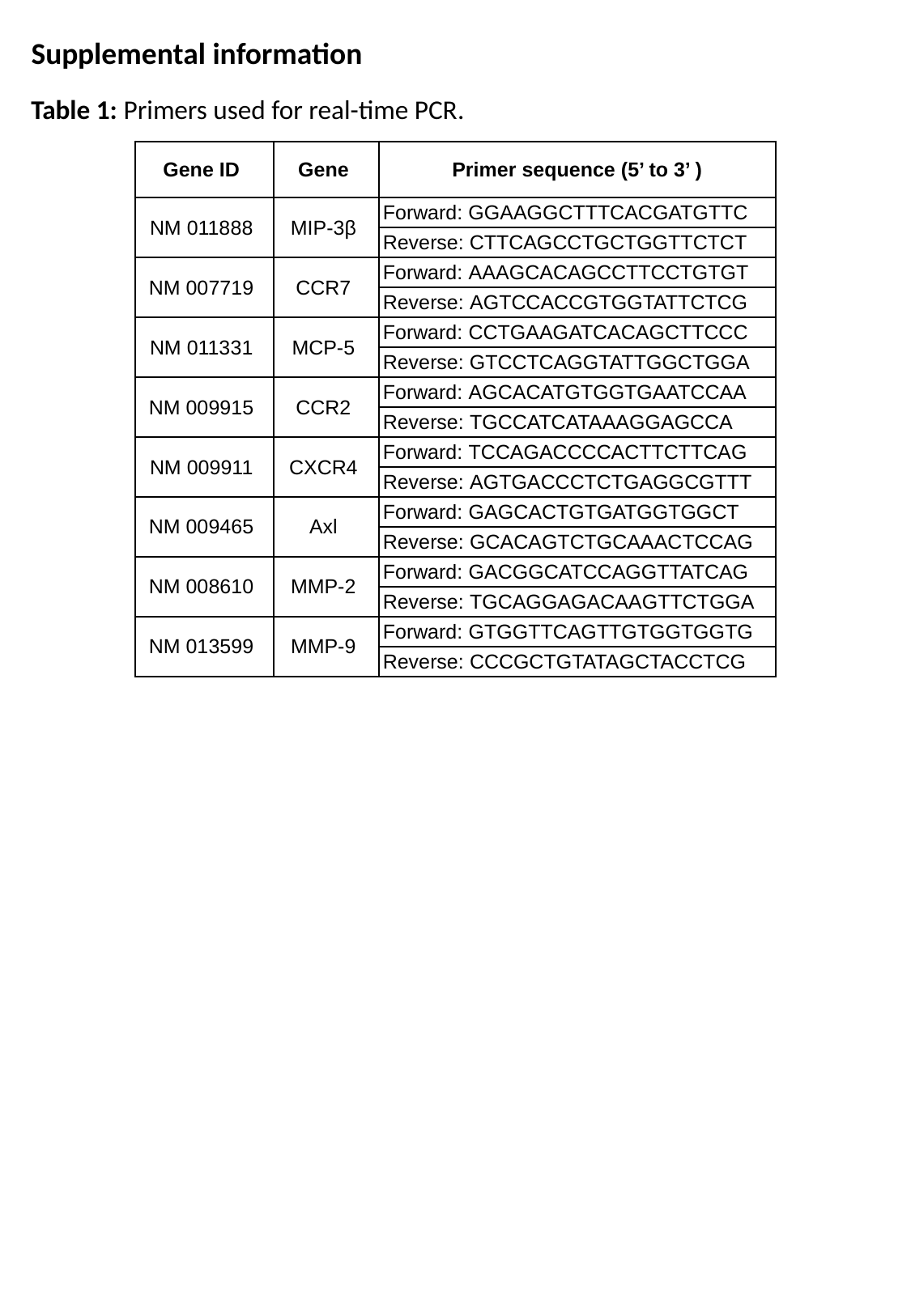

Supplemental information
Table 1: Primers used for real-time PCR.
| Gene ID | Gene | Primer sequence (5’ to 3’ ) |
| --- | --- | --- |
| NM 011888 | MIP-3β | Forward: GGAAGGCTTTCACGATGTTC |
| | | Reverse: CTTCAGCCTGCTGGTTCTCT |
| NM 007719 | CCR7 | Forward: AAAGCACAGCCTTCCTGTGT |
| | | Reverse: AGTCCACCGTGGTATTCTCG |
| NM 011331 | MCP-5 | Forward: CCTGAAGATCACAGCTTCCC |
| | | Reverse: GTCCTCAGGTATTGGCTGGA |
| NM 009915 | CCR2 | Forward: AGCACATGTGGTGAATCCAA |
| | | Reverse: TGCCATCATAAAGGAGCCA |
| NM 009911 | CXCR4 | Forward: TCCAGACCCCACTTCTTCAG |
| | | Reverse: AGTGACCCTCTGAGGCGTTT |
| NM 009465 | Axl | Forward: GAGCACTGTGATGGTGGCT |
| | | Reverse: GCACAGTCTGCAAACTCCAG |
| NM 008610 | MMP-2 | Forward: GACGGCATCCAGGTTATCAG |
| | | Reverse: TGCAGGAGACAAGTTCTGGA |
| NM 013599 | MMP-9 | Forward: GTGGTTCAGTTGTGGTGGTG |
| | | Reverse: CCCGCTGTATAGCTACCTCG |

## Slide 2
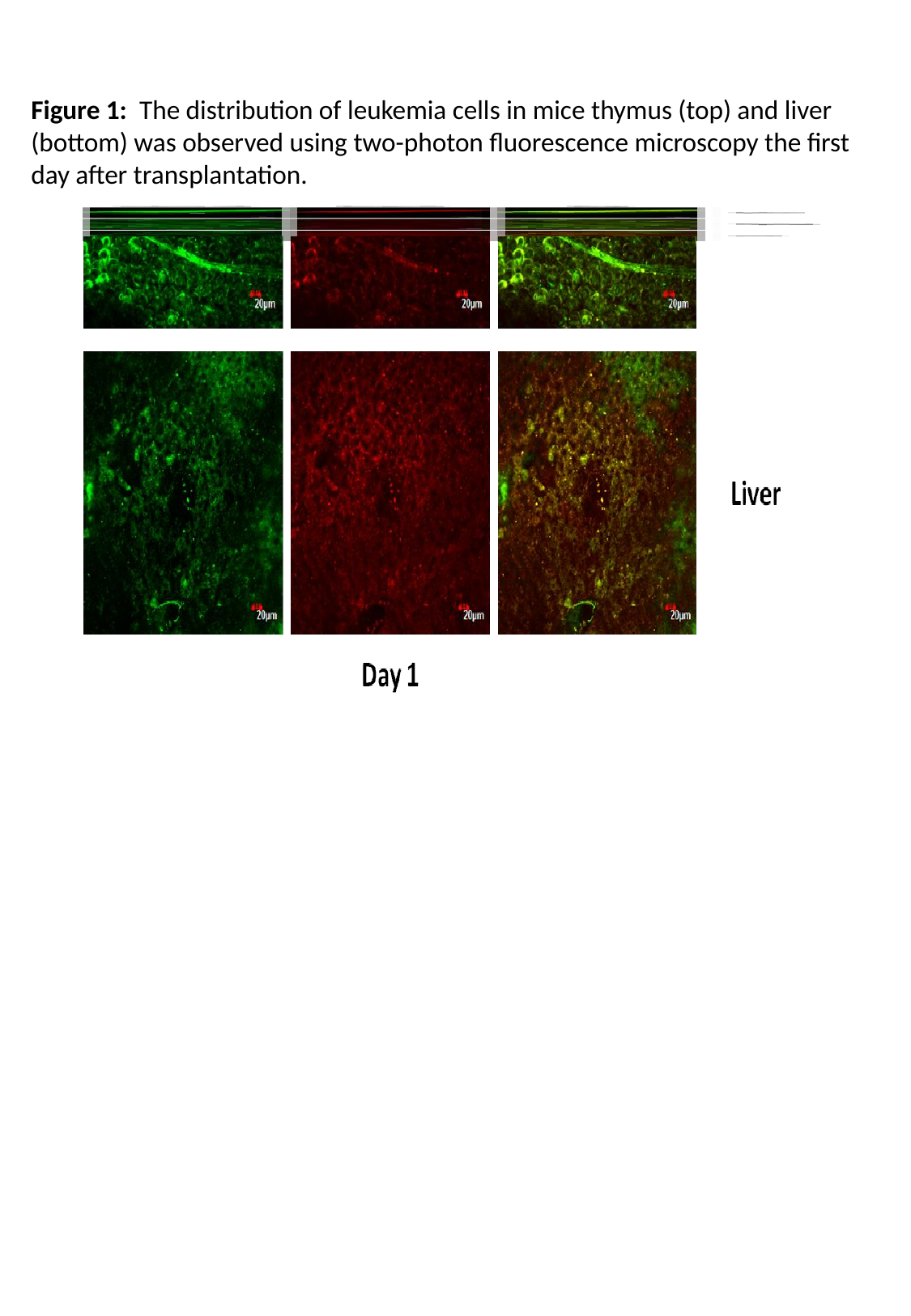

Figure 1: The distribution of leukemia cells in mice thymus (top) and liver (bottom) was observed using two-photon fluorescence microscopy the first day after transplantation.
